# The German Music@Home: Validation of a questionnaire measuring at home musical exposure and interaction of young children

**DOI:** 10.1371/journal.pone.0235923

**Published:** 2020-08-10

**Authors:** Nora K. Schaal, Nina Politimou, Fabia Franco, Lauren Stewart, Daniel Müllensiefen

**Affiliations:** 1 Institute of Experimental Psychology, Heinrich-Heine-University, Duesseldorf, Germany; 2 Department of Psychology and Human Development, University College London, London, United Kingdom; 3 Department of Psychological Sciences, Birkbeck University of London, London, United Kingdom; 4 Department of Psychology, Middlesex University, London, United Kingdom; 5 Department of Psychology, Goldsmiths University of London, London, United Kingdom; 6 University of Music, Drama and Media, Hanover, Germany; Università degli Studi di Perugia, ITALY

## Abstract

The present study introduces the German version of the original version of the Music@Home questionnaire developed in the UK, which systematically evaluates musical engagement in the home environment of young children. Two versions are available, an Infant version for children aged three to 23 months and a Preschool version for children aged two to five and a half years. For the present study, the original Music@Home questionnaire was translated from English into German and 656 caregivers completed the questionnaire online. A confirmatory factor analysis showed moderate to high fit indices for both versions, confirming the factor structure of the original questionnaire. Also, the reliability coefficients for the subscales (Parental beliefs, Child engagement with music, Parent initiation of singing, Parent initiation of music-making for the Infant version and Parental beliefs, Child engagement with music, Parent initiation of music behavior and Breadth of musical exposure for the Preschool version) ranged from moderate to high fits. Furthermore, the test-retest analysis (*N* = 392) revealed high correlations for the general factor and all subscales confirming their internal reliability. Additionally, we included language questionnaires for children of two and three years of age. Results showed that higher scores on the Music@Home questionnaire were moderately associated with better language skills in two-year-olds (*N* = 118). In sum, the study presents the validated German Music@Home questionnaire, which shows good psychometric properties. The two versions of the questionnaire are available for use in order to assess home musical engagement of young children, which could be of interest in many areas of developmental research.

## Introduction

Music has always been an integral part of human life and positive effects of musical training and interventions on social, cognitive and health aspects have been reported in numerous research studies [1–5]. Research has also shown that across cultures, children are exposed to music from an early age [6, 7] and the positive effects of music on children’s development and performance on different skills have been shown and critically discussed [8, 9].

Until now, most studies investigating the impact of music on children’s development and cognitive performance have concentrated on formal musical training. For example, cross-sectional studies have shown that children with formal musical training exhibit better performance in cognitive functions such as dual task performance and exhibit higher intelligence scores [10–12] compared to children without musical training. Furthermore, intervention studies have shown that linguistic functions such as phonological awareness, pitch discrimination and speech segregation can be improved with short formal musical training programs [13–16]. In these research studies the amount of formal musical training has been the main measure and children are often categorized as “musicians” (with formal musical training) and “non-musicians” (no formal musical training) based on this variable [11, 12].

However, research has also shown that musical engagement of a child goes beyond musical training, as music is often part of the home environment. Music is often embedded in daily routines and is frequently used to interact with young children [17, 18]. Furthermore, it has been shown that music can play an important part in everyday activities of under-fives [19].

Recently, Kreutz and Feldhaus [20] examined the development of familial musical engagement as well as other everyday activities, such as reading and shopping and how these are related to children’s personality. They revealed that the amount of musical activity declined in this age range as children grew older (ages ranged from seven to 14 years), in contrast to other activities which tended to remain stable over time. Furthermore, the results highlighted that musical engagement (singing and playing musical instruments) was associated with the personality factors Prosocial Behaviour, Intimacy and Admiration (i.e. appreciation). Overall, the study showed that music activities in the family setting have an effect on family dynamics, relationships and children’s personality [20], emphasizing that musical engagement at home is an important factor to consider when conducting studies with children.

In recent years a few questionnaires have been developed in order to evaluate musicality not only as a reflection of individuals’ formal musical training, but as a multifaceted and comprehensive construct in adult populations such as the *Goldsmiths Musical Sophistication Index* (Gold-MSI, [21]) and the *Music Use and Background Questionnaire* (MUSEBAQ, [22]). These questionnaires typically have a multi-dimensional structure with several subscales and an overall score. The questionnaires have been well received by the music research community and musical background/ expertise as measured by these questionnaires have been important covariates in many studies [i.e. 23, 24–26]. Moreover, several translations of musical background questionnaires have been published [27–30], which enables international comparisons of research results.

In addition, a number of questionnaires were developed to assess musical exposure of children at home. Already in 1985, the Home Musical Environment Scale (HOMES) was introduced which is a parent self-report questionnaire that evaluates parent-child musical engagement of school-aged children on four factors (i.e. parents’ attitude towards music and musical involvement with child, parental concert attendance, parent-child ownership and use of records/tapes, parent plays musical instrument) [31]. Comparable to the HOMES which was developed for music education purposes, the Children’s Music-Related Behavior Questionnaire (CMRBQ, [32]) was developed in order to evaluate musical behaviors and needs of under-fives in order to integrate these into preschool education settings. The CMRBQ comprises eight factors of which seven focus on the child’s behaviour (Attention & Emotion, Vocalizations, Moving, Daily Routines, Requests, Taking Turns, Creativity) and the eighth factor evaluates Parent Music Activities. Additionally, in 2018 Cogo-Moreira and Lamont [33,34] introduced the *Exposure to Music in Childhood Inventory*, a questionnaire which was developed to be suitable for children and assess their exposure to musical activities and behavior. The questionnaire comprises two factors: the first factor covers personal musical experiences such as music listening, home musical environment and the impact of television and the internet, whereas the second factor covers social elements such as playing an instrument, active and public music activities and performing [33]. The questionnaire is suitable for children aged five to thirteen years and the authors emphasize that the questionnaire evaluates the amount and type of musical activities, which go beyond musical training and the dichotomous categorization into non-musicians and musicians.

### The English Music@Home questionnaire

In 2018, Politimou and colleagues introduced the Music@Home questionnaire, a parent-report instrument that evaluates musical engagement in the home environment focusing on infants and young children. The Music@Home enriched the available range of questionnaires by concentrating on an age group that had so far lacked a systematic measurement for the home musical environment. Two different versions are available, one for infants (aged three to 23 months) and one for preschoolers (aged two to five and a half years). The Infant version includes 18 items, whereas the Preschool version comprises 17 items, and responses are given on a seven-point Likert scale ranging from completely agree to completely disagree. For the two versions of the Music@Home questionnaire a general factor can be calculated as well as scores on different subscales. These are Parental beliefs, Child engagement with music, Parent initiation of singing, and Parent initiation of music-making for the Infant version and Parental beliefs, Child engagement with music, Parent initiation of musical behavior and Breadth of musical exposure for the Preschool version. The confirmatory fit indices showed moderate to good fit for both versions (CFI of .872 for the Preschool version and .963 for the Infant version) and the test-retest analysis revealed high correlations (.65 to .87). Furthermore the study demonstrated that the Music@Home scores showed moderate but significant correlations (.24 to .53) with the Children’s Music Behavior Inventory [32] highlighting convergent validity of the questionnaire. Furthermore, the Music@Home showed significant associations with musical background of the parents as assessed with the Musical Training and Active Engagement scales of the Goldsmiths Musical Sophistication Index [21].

### Aim of the present study

As no German questionnaire exists for the evaluation of the home musical environment of young children, the primary purpose of the present study was to translate and adapt the Music@Home into German following standard recommendations from the literature. A secondary aim was to validate the questionnaire (Infant and Preschool version) by testing whether the factor structure of the original English Music@Home questionnaire could be replicated with a German sample. In addition, we tested the internal and test-retest reliability of the questionnaire. Finally, in order to take a first step towards exploring the utility of the German Music@Home questionnaire in general developmental research, the associations between the Music@Home scores with parents’ musical engagement and children’s language development were investigated in a subset of the sample.

## Methods

### Participants

A total of 656 caregivers (616 mothers and 40 fathers; mothers and fathers were from different families) participated in this online study between November 2018 and February 2019. Inclusion criteria for the study were that participants had a child aged between 3 months and 5 ½ years and spoke German sufficiently well to fill in the questionnaire. The sample was obtained through convenience sampling and is therefore limited regarding its representativeness. As presented in Table 1, the mean age of the caregivers filling out the questionnaire was 37.5 years (*SD* = 4.5 years) with a range from 25 to 51 years. The mean age of the child they filled out the questionnaire for was 2.3 years (*SD* = 1.3 years; range: 3month to 5 ½ years). All participants lived in Germany and 581 participants (88.6%) indicated that German was their native language whereas the remaining 75 caregivers (11.4%) had another mother tongue but indicated that they spoke German fluently. An overview of demographic details of the sample is presented in Table 1. From our overall sample of 656 caregivers, 326 (313 mothers and 13 fathers) filled in the questionnaire for their child aged between 3 and 24 months and therefore filled in the Infant version of the Music@Home questionnaire and 330 (303 mothers and 27 fathers) filled in the Preschool version for their child aged between 2 and 5 ½ years.

**Table 1:** Overview of relevant demographic details of the sample.

	M (SD)	n	%
**Age**	37.5 (4.5)		
**Gender**			
Female		616	93.9
Male		40	6.1
**School Education** (n = 3 missing values)			
Did not complete school qualification		/	/
School Qualification with 14 years of age		6	1.0
First School Qualification (e.g. Realschulabschluss/GCSE)		44	6.7
Second school qualification (e.g. Abitur/ A-levels)		603	92.3
**Level of monthly family income** (n = 55 missing values)			
< 1750 Euro		26	4.0
1750–3500 Euro		125	19.1
3500–5000		208	31.7
> 5000 Euro		242	36.9

For the test-retest analysis 392 caregivers (60% of the original sample) participated again in the study. Two-hundred-one caregivers filled in the Infant questionnaire (62% from the original sample) and 191 caregivers filled in the Preschool version (58% from the original sample). In 15 cases the caregivers did not fill in the same version of the questionnaire (i.e. as the child turned two in between the two measurement time points) and were therefore excluded for the test-retest analysis. The study was approved by the ethics committee of the Heinrich-Heine-University in Düsseldorf. Participants gave informed written consent.

### Materials

#### Music@Home questionnaire

The self-report Music@Home questionnaire comprises two versions evaluating the home musical environment: the Infant version for children of three to 24 months, and the Preschool version, which should be used for children between two and five and a half years. The Infant version contains 18 items and the Preschool version 17 items. For all items a 7-point agreement scale is used, ranging from 1 = completely disagree to 7 = completely agree. For negatively worded items reverse coding was used. The Infant version comprises four subscales, namely Parental beliefs (4 items), Child engagement with music (6 items), Parent initiation of singing (5 items) and Parent initiation of music-making (3 items). The Preschool version also has a four-factor underlying structure with the subscales Parental beliefs (5 items), Child engagement with music (4 items), Parent initiation of musical behavior (4 items) and Breadth of musical exposure (4 items). The factor structures of both Infant and Preschool versions showed moderate to good fit when the questionnaires were administered to a UK sample (for more detailed information see [34]).

#### Child language questionnaire

In order to evaluate language skills, two versions of the *Sprachbeurteilung durch Eltern* (SBE; language assessment through parents; [35]) questionnaire were used, which are questionnaires that are used at routine medical check-ups in Germany to evaluate language development. Two different versions were used. The SBE-2 is applied at the medical U6 check-up when the child is approximately two years old (20 to 26 months) and the SBE-3 is applied at the U7 check-up when the child is around three years old (32 to 36 months) (Note: The “U” examinations are compulsory check-ups every child in Germany needs to complete with caregiver at a pediatrician. They start with the first examination (U1) at birth and end with the U9 when children are five years old).

The SBE-2 and the SBE-3 were originally developed in order to identify developmental language delays, but the validity for also evaluating language development and delay in research studies has been shown [36–38]. For the SBE-2 caregivers need to indicate which words their child can already say from a list of 57 words and additionally there is one yes/no grammar question, which asks whether the child uses two-word phrases. The SBE-3 for three years olds contains a word list of 82 words and caregivers are asked to indicate which words their child speaks, in addition to 15 grammatical items. All items concentrate on speech production. For scoring, one point is given for every word the child is able to speak and for every grammar item the child uses correctly. For the SBE-2 the maximum score is 58 (57 word items and 1 grammar item). For the SBE-3 the number of correct grammar items (maximum 15) is multiplied by six and then the number of word items (maximum 82) is added and therefore the maximum score here is 172 [35].

#### Socioeconomic status and parental musical engagement

In order to evaluate the socioeconomic status of the caregiver who completed the questionnaire, we included questions regarding the highest school and academic education, as well as occupation and monthly household income.

Furthermore, in order to evaluate musical engagement of the caregiver, we included two dimensions of the Goldsmiths Musical Sophistication Index (Gold-MSI), [21, 27], namely Musical Training and Active Engagement. The Musical Training scale comprises seven items and the dimension Active Engagement nine items. Each item is rated on a seven-point likert scale and by adding all items belonging to the dimension respectively a sum score is generated. See Müllensiefen et al. [21] and Schaal et al. [27] for more information.

### Translation process

We translated the Music@Home questionnaires (Infant and Preschool version) following recommendations from the literature and used the same procedure established by previous scientific questionnaire translation studies [27, 30, 39, 40]. First the English questionnaires were translated into German from three independent persons who were all fluent in German and English. Two of them were German native speakers with excellent English skills and one of them had been brought up bilingually. Then, the three translations were compared with each other and discrepancies were resolved by the first author in close correspondence with the four translators. Next, the first German versions were developed and translated back into English by an English native speaker, who is also fluent in German. The back translated English versions were then compared to the original English Music@Home questionnaires and if required, the German items were adjusted in order to ensure that the items had the same meaning in both versions. The two versions of the German Music@Home questionnaire were then proofread by German colleagues, who checked correct spelling and style. The aim of the translation process was to receive a German version of the Music@Home questionnaires which are semantically, conceptually and culturally equivalent to the English versions [41]. The items of the two versions of the German Music@Home questionnaire are attached as Supportive Information alongside the original English items (S1 and S2 Data).

### Procedure

This online study was administered via the online platform www.soscisurvey.de. The link to the questionnaire was sent to approximately 1200 parents with children between the age of three months and five and a half years via email. The email addresses were taken from a database of the Babylab at the Heinrich-Heine-University.

The parents were asked to fill in the questionnaire online on a home computer and to fill in the survey for their youngest child. Informed written consent was obtained at the beginning of the online questionnaire. Informed written consent was obtained by participants ticking a box “I have read and understood the consent form and agree to take part in the online experiment” without entering other personal information.

The survey included demographic questions, the German Music@Home questionnaire, the items of the two Gold-MSI dimensions and the items regarding the socio-economic status. The participants received the appropriate version (Infant or Preschool) of the Music@Home questionnaire depending on the age of their youngest child. Additionally, if the child for whom the survey was filled out for was between 20 and 26 months of age (*N* = 118), the parents were directed to the SBE-2 language questionnaire and if the child was between 32 and 36 months of age (*N* = 99) the parents were asked to fill in the SBE-3 language questionnaire. At the end of the questionnaire the participants were asked if they would be willing to fill in parts of the questionnaire again in approximately two to four weeks, and if so, were requested to leave their email address. They were instructed that, if they leave an email address, their data no longer would be anonymous but would be saved with an identification code in order to link the data of the first and second part together. Overall, completion of the survey took 15 to 20 minutes.

All participants who gave their email address received a link to the second survey approximately 2 weeks (range: 2–6 weeks, mean time lag of filling in the questionnaires: 17 days ± 7 days) after first completion with a personal ID in order to match the data of the first and second measurement time point. The second survey only included the Music@Home questionnaire (either Infant or Preschool version). All participants who also completed the second survey had the chance to enter a prize draw to win one of five 20 Euro Amazon vouchers.

### Data analysis

In order to explore whether the factor structure of the German Music@Home questionnaires was similar to the factor structure of the original English versions, we applied the same analysis to the German data as Politimou et al. [34] applied to the English sample.

We applied a confirmatory factor analysis (CFA) in order to establish factorial validity of the Music@Home questionnaires using the R package lavaan [42]. For both Infant and Preschool versions, a bi-factor model was evaluated where the general factor impacted directly on all items (i.e., all items loading directly on the general factor) while the sub-factors also impacted on the items associated with them (i.e., individual items also loaded on their respective subfactor). Furthermore, scores for each Music@Home dimension as well as for the general factors were calculated by summing up the appropriate item scores. In order to calculate the internal reliability of each subscale of the Music@Home questionnaires as well as of the general Music@Home factors, we used three different measures (Cronbach’s alpha, MacDonald’s omega total, and Guttman’s lambda 6). For the test-retest reliability analysis Pearson correlations were calculated.

As a next step, we performed correlational analyses to assess convergent validity between the Music@Home questionnaires and the two dimensions (Musical Training and Active Engagement) of the Gold-MSI in order to test whether the Music@Home scores was associated with musical characteristics of the parents. Furthermore, for the appropriate sub-samples correlation analysis was performed between the Music@Home questionnaires and the two language questionnaires. Regarding the scores of the SBE2 and possible associations with the Music@Home scores, two sets of analysis had to be performed as approximately half (*N* = 67) filled in the Infant version as their child was 20–23 months old and the other half (*N* = 51) filled in the Preschool version as their child was 24–26 months old. Additionally, partial correlations were calculated between Music@Home scores and language scores when controlling for parental school education. We report the p-values of the correlations without correction of multiple comparisons and state confidence intervals as it has been argued recently that effect sizes and their confidence intervals are more meaningful for interpretation than p-values, even if corrected for multiple testing [43].

The influence of SES variables was checked separately by performing Spearman correlations between parental school education and family income and general factors of the Infant and Preschool versions as well as the language questionnaires SBE2 and SBE3.

## Results

**Fig 1:**
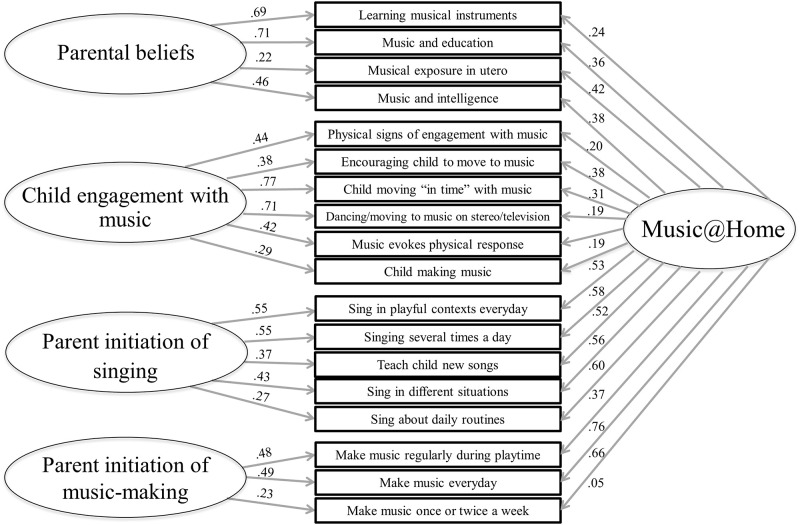
Factor structure and item loadings of the Music@Home Infant questionnaire.

**Fig 2:**
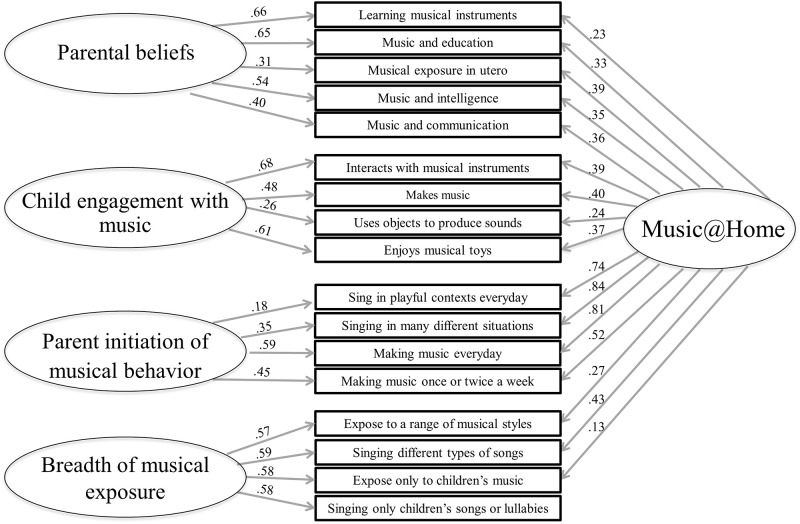
Factor structure and item loadings of the Music@Home Preschool questionnaire.

The results of the confirmatory factor analyses for both versions are presented in Table 2. The Music@Home Infant and Preschool version show good fit indices, confirming the factor structure of the English version. The factor structure and item loadings are presented in Fig 1 for the Infant version and in Fig 2 for the Preschool version.

**Table 2:** Confirmatory factor analysis for the Preschool and Infant version of the Music@Home questionnaire.

Models	χ2	df	RMSEA	CFI	TLI	SRMR
M@H Infant	183.29	117	.042	.962	.951	.042
M@H Preschool	259.68	104	.067	.908	.880	.063

Regarding reliability of the questionnaires, moderate to high coefficients were obtained for the general factors and subscales of both the Infant and Preschool versions (see Table 3). For the subscale Parent initiation of music-making of the Infant questionnaire, Cronbachs α was slightly lower with .529. The corresponding omega coefficient was at an acceptable level (.68).

**Table 3:** Estimates of internal reliability (Cronbach’s alpha, MacDonald’s omega total, and Guttman’s lambda 6) and test-retest correlations for the general factors and subscales of the Infant and Preschool version of the German Music@Home questionnaire.

	alpha	omega.tot	G6	test-retest
Music@Home Infant General Factor	.807	.827	.850	.828
Parental beliefs	.642	.724	.600	.700
Child’s active engagement	.738	.758	.728	.703
Parent initiation of singing	.794	.817	.774	.797
Parent initiation of music-making	.529	.680	.566	.735
Music@Home Preschool General Factor	.822	.838	.863	.823
Parental beliefs	.714	.752	.691	.803
Child’s active engagement	.657	.723	.614	.663
Parent initiation of musical behaviors	.785	.796	.801	.702
Breadth of musical exposure	.679	.690	.629	.745

All test-retest correlations were significant with p < .001, uncorrected; *n* = 194 for the Infant version and *n* = 183 for the Preschool version

Test-retest correlations were high for the general factors of the Infant and Preschool questionnaire respectively (.828 and .823, both p values < .001) confirming good test-retest reliability for both versions of the German Music@Home. The individual subscales also revealed high test-retest correlations (see Table 3).

Regarding the associations between the Gold-MSI scores and the Music@Home questionnaires, the data revealed, for the Infant version, significant small to moderate correlations between Active Engagement of the Gold-MSI and all subscales and the general factor of the Music@Home (r-scores between .173 and .395) and between Musical Training of the Gold-MSI and all subscales, except Child’s active engagement, and the general factor of the Music@Home (r-scores between .188 and .316). Similarly, for the Preschool version, significant small to moderate correlations between Active Engagement of the Gold-MSI and all subscales and the overall score of the Music@Home (r-scores between .195 and .442) and between Musical Training of the Gold-MSI and all subscales, except Breadth of musical exposure, and the general factor of the Music@Home (r-scores between .177 and .304) were shown.

The subsample-analysis exploring associations between the Music@Home scores and language development showed moderate but significant correlations between the SBE-2 scores (children aged 20–26 months) and the general factor of both versions as well as between language development and most subscales of the Infant and Preschool version (see Table 4). In contrast, for children aged 32–36 months (N = 86) no associations were revealed between the SBE-3 language scores and Music@Home scores (p-values > .156). When performing partial correlations between the Music@Home scores and language development and controlling for parental highest education levels, the correlations showed the same patterns (Table 4) by revealing significant correlations between SBE-2 scores and the Music@Home scores, whereas the associations between SBE-3 and Music@Home scores turned out non-significant.

**Table 4:** Associations between the evaluated language skills of 20–26 month old children (SBE-2) and of 32–36 months old children (SBE-3) and the Music@Home scores (Infant and Preschool version).

	Infant version					Preschool version				
	GF	PB	CAE	PIS	PIM	GF	PB	CAE	PIMB	BME
SBE-2	.391*	.354*	.113	.342*	.264*	.364*	-.046	.335*	.300*	.432*
	[.158;.649]	[.089;.519]	[-.076; .375]	[.153;.517]	[.017;.433]	[.163;.594]	[-.284; .249]	[.109; .557]	[.114;.522]	[.046;.630]
	.414*	.368*	.*135*	.348*	.283*	.363*	*-*.*044*	.338*	.299*	.436*
	*[*.*247;*.*606]*	*[*.*175;*.*551]*	*[-*.*049;*.*369]*	*[*.*204;*.*533]*	*[*.*072;*.*490]*					
						*[*.*130;*.*604]*	*[-*.*346;*.*242]*	*[*.*050;*.*628]*	*[*.*023;*.*540]*	*[*.*143;*.*622]*
SBE-3	/	/	/	/	/	.001	-.156	-.011	-.004	.151
						[-.206;.216]	[-.356;.029]	[-.224;.215]	[-.270;.288]	[-.077;.439]
						.*012*	*-*.*132*	*-*.*017*	*-*.*001*	.*164*
						*[-*.*208;*.*316]*	*[*.*-339;*.*099]*	*[-*.*224;*.*220]*	*[-*.*255;*.*297]*	*[-*.*110;*.*404]*

* indicates that non-corrected p-values are < .05, when correcting for multiple comparisons the p-values are non-significant; the italic values are the r-scores of the partial correlations when controlling for parental highest education; confidence intervals are presented in parentheses.

*n* = 67 for SBE2 and Infant correlation, *n* = 51 for the SBE2 and Preschool version and *n* = 86 for the SBE3 and Preschool correlation.

GF: general factor; PB: Parental beliefs; CAE: Child’s active engagement; PIS: Parent initiation of singing; PIM: parent initiation of music-making; PIMB: parent initiation of musical behaviour; BME: breadth of musical exposure.

The correlations between the SES variables and the Music@Home scores were non-significant: no associations could be revealed between highest school qualification and the general factors of the Infant and Preschool version (*p* = .116 and .236) nor between family income and the general factors of both versions (*p* = .323 and .120).

Regarding associations between parental SES and the language scores, the correlations between school education and family income and SBE2 as well as the correlation between family income and SBE3 were non-significant, however the correlation between parental school education and language scores of the SBE3 in three year olds was significant with *r* = .224, *p* = .038.

## Discussion

The aim of the current study was to adapt the Infant and Preschool versions of the Music@Home questionnaire into German and validate the translated versions with German samples. The results revealed that both versions of the questionnaire (i.e., Infant and Preschool) showed acceptable to good confirmatory fit to the data from the German convenience sample. Furthermore, the Music@Home German questionnaires showed good internal and test-retest reliability. In sum, we were able to confirm that the factor structure of the original English Music@Home questionnaires can be reliably replicated with a German sample. Specifically, similarly to the English version, the present results confirm an overall factor as well as four subscales for the Infant and Preschool version respectively. However, the reliability analysis showed a divergence between the coefficients alpha and omega in the Parent initiation dimension of the Infant version, which warrants a comment here. This divergence can be explained by the fact that the different items on this subscale have loadings that are considerably different from each other. Hence, in practical application scenarios, it might be worth considering computing factor scores using regression of the Bartlett method instead of simple sum scores or averages for the Parent initiation subscale or all subscales of the Infant inventory because these alternative methods for factor scoring do not make the assumption of equal item loadings. Cronbach’s alpha is based on the assumption that all item of a scale are equally important and hence have the same weight for computing the coefficient. However, in practice this is rarely the case. In contrast, MacDonald’s omega allows differences in the importance of items and therefore item weights can differ in the computation of the coefficient.

The present study revealed positive associations between the two subscales of the Gold-MSI (Musical Training and Active Engagement) and the general factor and most subscales of the Music@Home Infant and Preschool questionnaires. The results are in accordance with the results by Politimou and colleagues [34] regarding the Infant version and only partly in accordance regarding the Preschool version as in the English sample only Active Engagement but not Musical Training correlated with the Music@Home scores. Overall, it seems that musical engagement of the child is influenced by the general level of parents’ musical activities and involvement with music, which is in accordance with previous research [44].

In the present study, we also included two versions of a language development parent-report questionnaire in order to examine relationships between home musical environment and language development of the children. The results show that Music@Home scores are significantly associated with language development scores in two-year-old children. However, no associations were revealed when considering the language scores of the three year olds. The results of the two years olds are interesting as they indicate that an enriched home musical environment can be associated with more rapid language development in young toddlers. A positive link between children’s musical skills and/or formal musical training and language development has also been reported in previous studies [15, 45, 46] and an association between higher frequency of musical interactions and enriched musical exposure and development of complex language skills has been reported in a study with 3- and 4-year-old children [47]. However, it is important to note that the results presented here need to be interpreted with caution. Even though the present data revealed no correlations between parental SES variables and reported language scores on the SBE2 of two year olds, many other confounding factors, which we have not controlled for, such as environmental and genetic elements may influence language development [48, 49]. Furthermore, the fact that no associations were present between Music@Home scores and language development in three year olds needs to be considered. There are several explanations for the different findings in the two and three year olds: (i) we used two different versions of language questionnaires for the assessment of language development in the two age groups, which lowers the internal validity and strength of comparison of the two age groups [50], (ii) evaluating language development in three year olds is more complex and therefore leads to higher variability among participants [51], (iii) many other influencing factors may overshadow possible effects in the three year olds such as language input of the parents and quality of child care [52] as children of this age are more likely to be attending a range of activities outside the home. Furthermore, the finding we present, that a higher parental school qualification is associated with better language scores of the three years olds, but not two-year olds, may indicate that the influence of socioeconomic factors on child development increases as children grow older [52]. In this respect, it is important to note that the parents, who participated in the current study, were mostly middle class, since we used a convenience sample in the present study. For future research, it would be important to try to recruit a more representative sample. The fact that our sample were predominantly middle-class parents may lead to a potential bias in communication and socialization strategies with their offspring [53]. The association between SES and parental education has been shown to be a strong predictor of early language development [54]. However, when separating SES/education from actual interaction variables, such as amount of daily verbal interaction with their infants, robust research has shown that the developmental outcome is predicted by the actual interaction variable rather than SES per se. For instance, Weisleder and Fernald [55] investigated a lower-SES sample and showed that differences in the amount of infant-directed verbal interactions mediated the infants’ abilities to process language hence predicted their expressive language at 24 months. Based on this perspective [56], we can speculate that the moderate associations between parental musical sophistication measures, such as formal training, and musical interactions with young children at home may well be mediated by SES. However, parent-infant musical interactions will be associated to individual differences independent of SES and the variations in the amount and quality of home music interactions that would predict child developmental outcomes.

Another limitation is that we did not include a measure for general parental engagement. It may be that parents who provide the two year old with a rich musical environment at home also engage in other forms of activities with the child such as reading which could influence language development. In future research, it would be desirable to also evaluate other home activities, next to musical engagement, in order to disentangle whether an overall enriched home environment leads to better language skills or whether language development is enhanced explicitly through musical engagement. More research looking at the relationship between musical engagement at home and language development is needed. It would be desirable to conduct a study with a larger sample of two and three year olds and include the Music@Home questionnaire, a language questionnaire such as the SBE-2 and SBE-3 and evaluate other forms of home activities in a follow-up study. However, the results of the present study indicate that it could be useful to assess home musical engagement in studies examining research questions on language acquisition and possibly other developmental areas and we suggest that the Music@Home questionnaire could be a useful tool for this.

In sum, this study presented the successful adaptation and validation of the German version of the Music@Home questionnaire, which can be used to measure musical engagement in the home beyond formal musical training in children under five. Both versions of the questionnaire displayed good psychometric properties, allowing researchers to reliably assess the home musical environment in two different age groups, and opening the way to novel research investigating the influence of early home musical experiences on a range of developmental outcomes. The English and German versions of the questionnaire are freely available for non-commercial research and can be obtained from the authors upon request.

## Supporting information

S1 Data Music@Home INFANT Version. (PDF)

S2 Data Music@Home PRESCHOOL Version. (PDF)
