# Supplementary material for: The German Music@Home: Validation of a questionnaire measuring at home musical exposure and interaction of young children
Source: PLoS One. 2020 Aug 10;15(8):e0235923. doi: 10.1371/journal.pone.0235923 (PMC7416926; doi:10.1371/journal.pone.0235923)
Supplement: S1 Data — (PDF) [file pone.0235923.s001.pdf]

Music@Home INFANT Version

Item	English Version	German Version
	**Parental beliefs**	**Überzeugungen der Eltern**
3	I believe that music is part of a well-rounded education	Ich finde, dass Musik Teil einer vielseitigen Erziehung ist.
12	My child was deliberately sung to/exposed to music whilst in the womb	Meinem Kind wurde im Mutterleib bewusst vorgesungen/Musik vorgespielt.
13	I believe music has an impact on my child's intelligence	Ich glaube, dass Musik sich auf die Intelligenz meines Kindes auswirkt.
18	I believe that children should learn to play an instrument	Ich finde, Kinder sollten lernen, ein Instrument zu spielen.
	**Child's active engagement**	**Aktive Beteiligung des Kindes**
6	My child displays no physical signs of engagement when there is recorded music on (e.g. bouncing or tapping)	Mein Kind zeigt keine physischen Anzeichen von Anteilnahme (z.B. Springen oder Klopfen), wenn Musik abgespielt wird.
7	Music does not evoke a physical response from my child	Musik ruft bei meinem Kind keine körperliche Reaktion hervor.
8	My child rarely makes music	Mein Kind macht selten Musik.
9	I encourage my child to move along to music	Ich ermutige mein Kind, sich zur Musik zu bewegen.
11	My child does not dance/move to music on the stereo or television	Mein Kind bewegt sich nicht zu Musik aus der Musikanlage oder dem Fernseher.
17	I have noticed my child moving in time with the beat of the music	Ich habe bemerkt, dass mein Kind sich im Takt der Musik bewegt.
	**Parent initiation of singing**	**Eltern initiiertes Singen**
1	I sing to/with my child several (e.g. 5 - 10) times a day	Ich singe mehrmals (z.B. 5 – 10 Mal) am Tag meinem Kind vor/mit meinem Kind
2	During our daily routine, I do not spend much time singing about what we are doing	In unserem Alltag verbringe ich nicht viel Zeit damit, über das zu singen, was wir gerade machen.
4	I sing to/with my child in many different situations (e.g. during playtime, with friends and family)	Ich singe für/mit mein(em) Kind in vielen verschiedenen Situationen (z.B. beim Spielen, mit Freunden und Familien).
15	I teach my child new songs	Ich bringe meinem Kind neue Lieder bei.
16	I sing in playful contexts to/with my child at least once a day	Ich singe mindestens einmal am Tag in spielerischen Kontexten für/mit mein(em) Kind.
	**Parent initiation of music-making**	**Eltern initiiertes Musikmachen**
5	I make music with my child (including toy instruments) almost everyday	Ich mache fast jeden Tag mit meinem Kind Musik (einschl. Spielzeuginstrumente).
10	I do not make music with my child (including toy instruments) more than once or twice per week	Ich mache nicht häufiger als ein- bis zweimal pro Woche mit meinem Kind Musik (einschl. Spielzeuginstrumente).
14	Making music with my child (including toy instruments) is a regular part of playtime at home	Mit meinem Kind Musik zu machen (einschl. Spielzeuginstrumente), gehört zum Spielen zuhause regelmäßig dazu

	**General Factor - Music at Home**	**Generalfaktor – Musik@Zuhause**
1	I sing to/with my child several (e.g. 5 - 10) times a day	Ich singe mehrmals (z.B. 5 – 10 Mal) am Tag meinem Kind vor/mit meinem Kind
2	During our daily routine, I do not spend much time singing about what we are doing	In unserem Alltag verbringe ich nicht viel Zeit damit, über das zu singen, was wir gerade machen.
3	I believe that music is part of a well-rounded education	Ich finde, dass Musik Teil einer vielseitigen Erziehung ist.
4	I sing to/with my child in many different situations (e.g. during playtime, with friends and family)	Ich singe für/mit mein(em) Kind in vielen verschiedenen Situationen (z.B. beim Spielen, mit Freunden und Familien).
5	I make music with my child (including toy instruments) almost everyday	Ich mache fast jeden Tag mit meinem Kind Musik (einschl. Spielzeuginstrumente).
6	My child displays no physical signs of engagement when there is recorded music on (e.g. bouncing or tapping)	Mein Kind zeigt keine physischen Anzeichen von Anteilnahme (z.B. Springen oder Klopfen), wenn Musik abgespielt wird.
7	Music does not evoke a physical response from my child	Musik ruft bei meinem Kind keine körperliche Reaktion hervor.
8	My child rarely makes music	Mein Kind macht selten Musik.
9	I encourage my child to move along to music	Ich ermutige mein Kind, sich zur Musik zu bewegen.
10	I do not make music with my child (including toy instruments) more than once or twice per week	Ich mache nicht häufiger als ein- bis zweimal pro Woche mit meinem Kind Musik (einschl. Spielzeuginstrumente).
11	My child does not dance/move to music on the stereo or television	Mein Kind bewegt sich nicht zu Musik aus der Musikanlage oder dem Fernseher.
12	My child was deliberately sung to/exposed to music whilst in the womb	Meinem Kind wurde im Mutterleib bewusst vorgesungen/Musik vorgespielt.
13	I believe music has an impact on my child's intelligence	Ich glaube, dass Musik sich auf die Intelligenz meines Kindes auswirkt.
14	Making music with my child (including toy instruments) is a regular part of playtime at home	Mit meinem Kind Musik zu machen (einschl. Spielzeuginstrumente), gehört zum Spielen zuhause regelmäßig dazu
15	I teach my child new songs	Ich bringe meinem Kind neue Lieder bei.
16	I sing in playful contexts to/with my child at least once a day	Ich singe mindestens einmal am Tag in spielerischen Kontexten für/mit mein(em) Kind.
15	I teach my child new songs	Ich bringe meinem Kind neue Lieder bei.
17	I have noticed my child moving in time with the beat of the music	Ich habe bemerkt, dass mein Kind sich im Takt der Musik bewegt.
18	I believe that children should learn to play an instrument	Ich finde, Kinder sollten lernen, ein Instrument zu spielen.
