# Supplementary material for: The German Music@Home: Validation of a questionnaire measuring at home musical exposure and interaction of young children
Source: PLoS One. 2020 Aug 10;15(8):e0235923. doi: 10.1371/journal.pone.0235923 (PMC7416926; doi:10.1371/journal.pone.0235923)
Supplement: S2 Data — (PDF) [file pone.0235923.s002.pdf]

Music@Home PRESCHOOL Version

Item	English Version	German Version
	**Parental beliefs**	**Überzeugungen der Eltern**
4	I believe that music is part of a well-rounded education	Ich finde, dass Musik Teil einer vielseitigen Erziehung ist.
9	I think musical activities are important for learning to communicate	Ich denke, dass musikalische Aktivitäten wichtig sind, um kommunizieren zu lernen
11	My child was deliberately sung to/exposed to music whilst in the womb	Meinem Kind wurde im Mutterleib bewusst vorgesungen/Musik vorgespielt.
12	I believe music has an impact on my child's intelligence	Ich glaube, dass Musik sich auf die Intelligenz meines Kindes auswirkt
15	I believe that children should learn to play an instrument	Ich finde Kinder sollten lernen ein Instrument zu spielen.
	**Child's active engagement**	**Aktive Beteiligung des Kindes**
2	My child enjoys making sounds/interacting with musical instruments (including toy ones)	Meinem Kind macht es Spaß, Geräusche zu machen/mit Musikinstrumenten zu interagieren (einschl. Spielzeuginstrumente).
5	My child enjoys toys with musical features	Mein Kind hat Freude an Spielzeug mit musikalischen Eigenschaften.
8	My child rarely makes music	Mein Kind macht selten Musik.
16	My child does not use objects to intentionally produce sounds	Mein Kind benutzt Gegenstände nicht, um absichtlich Geräusche zu erzeugen.
	**Parent initiation of musical behavior**	**Eltern initiiertes musikalisches Verhalten**
1	I sing to/with my child in many different situations (e.g. during playtime, with friends and family)	Ich singe für/mit mein(em) Kind in vielen verschiedenen Situationen (z.B. beim Spielen, mit Freunden und Familien)
10	I do not make music with my child (including toy instruments) more than once or twice per week	Ich mache nicht häufiger als ein- bis zweimal die Woche mit meinem Kind Musik (einschl. Spielzeuginstrumente).
13	I sing in playful contexts to/with my child at least once a day	Ich singe mindestens einmal am Tag in spielerischen Kontexten für/mit mein(em) Kind.
14	I make music with my child (including toy instruments) almost everyday	Ich mache fast jeden Tag mit meinem Kind Musik (einschl. Spielzeuginstrumente).
	**Breadth of musical exposure**	**Stilistische Breite**
3	I sing all different types of songs to my child (e.g. adult songs, traditional folk songs)	Ich singe meinem Kind alle möglichen unterschiedlichen Arten von Liedern vor (z.B. Lieder für Erwachsene, Volkslieder).
6	I sing mostly children's songs or lullabies to or with my child	Ich singe hauptsächlich Kinderlieder oder Wiegenlieder für/mit mein(em) Kind
7	I would only expose my child to "children's music"	Ich würde mein Kind nur mit Kinderliedern in Berührung bringen.
17	My child is exposed to a broad range of musical styles at home (e.g. pop, rap, dance, classical etc)	Mein Kind ist zuhause einer großen Vielfalt an Musikstilen ausgesetzt (z.B. Pop, Rap, Tanzmusik, Klassik, etc.)

	General Factor - Music at Home	Generalfaktor – Musik@Zuhause
1	I sing to/with my child in many different situations (e.g. during playtime, with friends and family)	Ich singe für/mit mein(em) Kind in vielen verschiedenen Situationen (z.B. beim Spielen, mit Freunden und Familien)
2	My child enjoys making sounds/interacting with musical instruments (including toy ones)	Meinem Kind macht es Spaß, Geräusche zu machen/mit Musikinstrumenten zu interagieren (einschl. Spielzeuginstrumente).
3	I sing all different types of songs to my child (e.g. adult songs, traditional folk songs)	Ich singe meinem Kind alle möglichen unterschiedlichen Arten von Liedern vor (z.B. Lieder für Erwachsene, Volkslieder).
4	I believe that music is part of a well-rounded education	Ich finde, dass Musik Teil einer vielseitigen Erziehung ist.
5	My child enjoys toys with musical features	Mein Kind hat Freude an Spielzeug mit musikalischen Eigenschaften.
6	I sing mostly children's songs or lullabies to or with my child	Ich singe hauptsächlich Kinderlieder oder Wiegenlieder für/mit mein(em) Kind
7	I would only expose my child to "children's music"	Ich würde mein Kind nur mit Kinderliedern in Berührung bringen.
8	My child rarely makes music	Mein Kind macht selten Musik.
9	I think musical activities are important for learning to communicate	Ich denke, dass musikalische Aktivitäten wichtig sind, um kommunizieren zu lernen
10	I do not make music with my child (including toy instruments) more than once or twice per week	Ich mache nicht häufiger als ein- bis zweimal die Woche mit meinem Kind Musik (einschl. Spielzeuginstrumente).
11	My child was deliberately sung to/exposed to music whilst in the womb	Meinem Kind wurde im Mutterleib bewusst vorgesungen/Musik vorgespielt.
12	I believe music has an impact on my child's intelligence	Ich glaube, dass Musik sich auf die Intelligenz meines Kindes auswirkt
13	I sing in playful contexts to/with my child at least once a day	Ich singe mindestens einmal am Tag in spielerischen Kontexten für/mit mein(em) Kind.
14	I make music with my child (including toy instruments) almost everyday	Ich mache fast jeden Tag mit meinem Kind Musik (einschl. Spielzeuginstrumente).
15	I believe that children should learn to play an instrument	Ich finde Kinder sollten lernen ein Instrument zu spielen.
16	My child does not use objects to intentionally produce sounds	Mein Kind benutzt Gegenstände nicht, um absichtlich Geräusche zu erzeugen.
17	My child is exposed to a broad range of musical styles at home (e.g. pop, rap, dance, classical etc)	Mein Kind ist zuhause einer großen Vielfalt an Musikstilen ausgesetzt (z.B. Pop, Rap, Tanzmusik, Klassik, etc.)
